# Early Predictors of Employment Status One Year Post Injury in Individuals with Traumatic Brain Injury in Europe

**DOI:** 10.3390/jcm9062007

**Published:** 2020-06-26

**Authors:** Juan Carlos Arango-Lasprilla, Marina Zeldovich, Laiene Olabarrieta-Landa, Marit Vindal Forslund, Silvia Núñez-Fernández, Nicole von Steinbuechel, Emilie Isager Howe, Cecilie Røe, Nada Andelic, CENTER-TBI Participants and Investigators

**Affiliations:** 1 Biocruces Bizkaia Health Research Institute, 48903 Barakaldo, Spain; silay60@gmail.com; 2 IKERBASQUE Basque Foundation for Science, 48013 Bilbao, Spain; 3 Department of Cell Biology and Histology, University of the Basque Country UPV/EHU, 48940 Leioa, Spain; 4 Institute of Medical Psychology and Medical Sociology, University Medical Center Göttingen, 37073 Göttingen, Germany; nvsteinbuechel@med.uni-goettingen.de; 5 Departamento de Ciencias de la Salud, Universidad Pública de Navarra, 31006 Pamplona, Spain; laieneolabarrieta@gmail.com; 6 Department of Physical Medicine and Rehabilitation, Oslo University Hospital, 0424 Oslo, Norway; mavfor@ous-hf.no (M.V.F.); e.i.howe@medisin.uio.no (E.I.H.); cecilie.roe@medisin.uio.no (C.R.); nandelic@online.no (N.A.); 7 Institute of Clinical Medicine, Faculty of Medicine, University of Oslo, 0318 Oslo, Norway; 8 Institute of Health and Society, Research Centre for Habilitation and Rehabilitation Models and Services (CHARM), Faculty of Medicine, University of Oslo, 0318 Oslo, Norway

**Keywords:** traumatic brain injury, prospective studies, multicenter studies, rehabilitation, outcome assessment, employment

## Abstract

Sustaining a traumatic brain injury (TBI) often affects the individual’s ability to work, reducing employment rates post-injury across all severities of TBI. The objective of this multi-country study was to assess the most relevant early predictors of employment status in individuals after TBI at one-year post-injury in European countries. Using a prospective longitudinal non-randomized observational cohort (The Collaborative European NeuroTrauma Effectiveness Research in TBI (CENTER-TBI) project), data was collected between December 2014–2019 from 63 trauma centers in 18 European countries. The 1015 individuals who took part in this study were potential labor market participants, admitted to a hospital and enrolled within 24 h of injury with a clinical TBI diagnosis and indication for a computed tomography (CT) scan, and followed up at one year. Results from a binomial logistic regression showed that older age, status of part-time employment or unemployment at time of injury, premorbid psychiatric problems, and higher injury severity (as measured with higher Injury severity score (ISS), lower Glasgow Coma Scale (GCS), and longer length of stay (LOS) in hospital) were associated with higher unemployment probability at one-year after injury. The study strengthens evidence for age, employment at time of injury, premorbid psychiatric problems, ISS, GCS, and LOS as important predictors for employment status one-year post-TBI across Europe.

## 1. Introduction

Traumatic brain injury (TBI) is one of the leading causes of disability and death worldwide [1,2]. Every year millions of individuals around the world suffer from TBI [3]. In those who survive, injuries are often associated with the presence of physical, cognitive, and emotional difficulties [4] in the short and long term. These TBI-related impairments generate problems with personal, social, family, and work reintegration following injury [4,5].

Past studies have reported employment rates that range from 8–72% in individuals with TBI during the first year after the injury [6,7,8]. Factors influencing employment status include age at the time of trauma [9,10,11,12,13,14], gender [9,12,15,16,17], ethnicity [17], marital or partner relationship status prior to injury [9,15,18,19,20], education level [8,10,13,19,20,21,22], previous employment status [8,10,13,15,18,21,23,24,25], occupation type [15,18,26], severity of the injury [8,11,13,14,15,18,23,27,28,29,30], cognitive functioning [11,14,16,27,31], depression status [27], and having autonomy in transportation [16,19,27,32,33].

Knowledge regarding factors that are associated with employment status in individuals with TBI one year after the injury is of great importance in order to develop and implement early rehabilitation programs and design vocational rehabilitation with appropriate follow-ups to ensure successful and lasting return to the workplace. However, the majority of studies on employment status have been carried out in non-European countries [9,20,21,27,34]. European countries exhibit important differences geographically, with respect to their populations, economically and mainly, in the type of health and social care system as compared to Non-European countries. The European health systems guarantee universal health care access and sickness benefits regardless of the level of income [35,36]; access that is not readily available for individuals with insufficient financial resources in countries outside Europe.

In Europe, 2.5 million individuals sustain a TBI every year [1,37] and the economic costs of the treatment of these patients exceed approximately 49.7 billion USD annually [1], which generates high expenses for the health systems of these countries. In addition, many of these individuals never return to work, which also increases economic costs due to loss of labor productivity. It is important to study early predictive factors involved in employment status following TBI in European countries to contribute to the development and implementation of future work reintegration and employment support programs for individuals with TBI in a universal health care system setting.

Hence, the objective of this study was to assess early relevant predictors of employment status in individuals hospitalized with TBI one year after the injury in European countries. This multi-country study, including all TBI severities, is part of the Collaborative European NeuroTrauma Effectiveness Research in TBI (CENTER-TBI) project [38]; thus, it should be possible to generalize the results to various geographical regions and populations.

## 2. Materials and Methods

### 2.1. Study Design and Participants

Data were collected within the CENTER-TBI project, from December 2014 to December 2019. CENTER-TBI is a prospective longitudinal non-randomized observational study across the severity spectrum of TBI from 63 centers in 18 European countries. Data were retrieved from the CENTER-TBI database using the data access tool Neurobot and core 2.0 sample (dataset frozen in May 2019). Inclusion criteria for the core data set were: (1) a clinical diagnosis of TBI, (2) indication for computed tomography (CT) scan, (3) enrollment within 24 h after injury, and (4) consent for study participation. The core dataset included three strata that were differentiated according to care paths: patients seen in the Emergency Room (ER); patients admitted to the intensive care unit (ICU); and patients primarily admitted to the hospital ward (ADM).

Patients with a severe pre-existing neurological disorder were excluded as such conditions may bias outcome assessments. For more details, see Maas et al. [38] and Steyerberg et al. [39]. The complete core data set consisted of *N* = 4509 individuals. According to the study design, patients admitted to the emergency room (ER) stratum were not interviewed one-year post TBI; thus, 3661 patients admitted to the ward or the ICU stratum are the subject of the current report.

**Figure 1:**
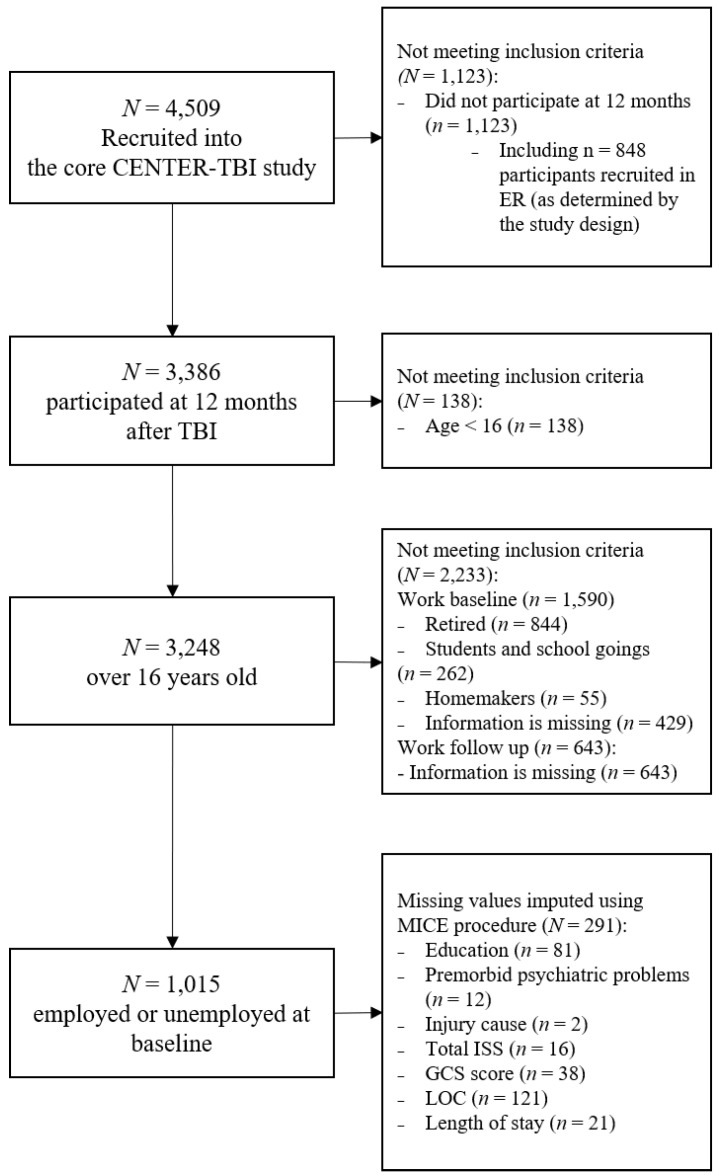
Sample flow chart diagram. Abbreviations: *N* = number (*n* = number in subgroups); ISS = Injury severity score; GCS = Glasgow Coma Scale; LOC = Loss of consciousness; Length of stay = Length of stay in the hospital.

The present study had the following inclusion criteria: (1) potential labor market participant (excluding retired persons, students, and homemakers), (2) 16 years of age or older, and (3) participated in the study at one-year follow-up. From the core data set, *N* = 1015 individuals met the study inclusion criteria; for details, see the sample flow chart in Figure 1.

### 2.2. Instruments and Variables

Employment status. Employment status was assessed at baseline and at follow up at 12 months after the injury. For the purpose of analysis, employment status before injury was recoded into three groups at baseline: *employed full-time* (i.e., working ≥ 35 h per week); *employed part-time* (i.e., working 20–24 h/week, ≤ 20 h/week, currently on sick leave, or in sheltered employment); or *unemployed* (unemployed or unable to work). One year after TBI, the employment status was collapsed into two groups: *employed* (including return to previous job, also with less hours, change of job, or sheltered employment) and *unemployed* (including unemployed or unable to work).

Sociodemographic variables. The following sociodemographic variables were collected at the time of injury: sex (*male* or *female*), age in years, marital status (*single* or *partnered* (*married, cohabitating*), education (*post-high school* (including individuals currently in diploma or degree-oriented program, or post-high school training), *secondary/high-school*, or *only primary school/others*).

The presence of premorbid psychological/psychiatric problems (*yes* or *no*) was characterized by anxiety, depression, sleep disorders, schizophrenia, drug abuse, or other psychiatric problems in the medical history of patients.

Injury related factors. The following injury-related variables were collected in the acute phase: cause of injury, TBI severity assessed by GCS, Injury Severity Score (ISS), loss of consciousness (LOC), and length of stay in hospital (LOS). For the present study, recruitment occurred in two strata according to care pathways: Admission stratum (ADM) or intensive care (ICU) stratum.

The cause of injury was collapsed into three groups: *road traffic accident*, *fall*, or *violent injury* (violence/assault, act of mass violence, or suicide attempt) combined with *other causes*.

TBI severity was evaluated with the Glasgow Coma Scale (GCS [40]). The score ranges from 3–15 (a higher score indicates a higher level of responsiveness), with 15 representing normal level.

The Injury Severity Score (ISS), an indicator of overall trauma severity, was calculated as the sum of the squares of three highest values of the Abbreviated Injury Scale score (AIS) [41] from different body regions. The ISS ranges from 0–75 (a higher score indicates greater severity of the trauma).

Loss of consciousness (LOC) covered three categories: *yes* (including suspected LOC), *no*, or *unknown*. The *unknown* LOC was treated as missing. The assessment was based on self-report, clinical interview, or the medical chart.

Length of stay (LOS) in the hospital was determined in days beginning with enrollment. It was derived using the information of the date and time of arrival at the study hospital and date and time of hospital discharge. Due to the skewed distribution, the variable was logarithmized for further analyses.

### 2.3. Ethical Approval

The CENTER-TBI study (EC grant 602150) has been conducted in accordance with all relevant laws of the EU if directly applicable or of direct effect and all relevant laws of the country where the recruiting sites were located. For further information on ethical approval, see https://www.center-tbi.eu/project/ethical-approval. Informed consent was obtained for all patients by the patients themselves and/or the legal representative/next of kin.

### 2.4. Statistical Analyses

Analyses proceeded according to the following strategy: (1) estimation of the initial theory-based model, (2) backward selection of the predictors based on a liberal *p*-value (*p* < 157) to determine factors contributing significantly to the model fit, and (3) estimation and internal validation of the final model.

To examine the predictors of employment status one year after TBI, a binomial logistic regression was performed. Employment status (employed/unemployed) one-year post-injury served as the dependent variable, with prediction of the probability of employment. The independent variables were derived from previously published research described in the introduction and were grouped as follows: sociodemographic and premorbid factors (sex, age, marital status, education, employment status at time of injury, and premorbid psychiatric problems), and injury related factors (stratum, injury cause, GCS, ISS, presence of LOC, and LOS in the hospital).

The amount of missing values varied from <0.01% (injury cause) to 11.9% (LOC). Missingness at random was assumed, and values were imputed using the multiple imputation by a chained equations procedure (MICE [42]) using the mice-package in R [43]. Overall model performance was assessed by performing a bootstrapping validation on imputed data and assessing optimism-corrected Nagelkerke’s R² [44] and area under the curve (AUC) (e.g., Reference [45]). Nagelkerke’s R² ranges from 0–1, demonstrating the relative information gain of the estimated model compared to a null model, which contains only the intercept without any predictors. The higher the Nagelkerke’s R², the better the model. The AUC assesses how well the model can distinguish between the groups of the dependent variable. The higher the AUC, the better the ability of the model to distinguish between employed and unemployed groups one year after TBI. For optimism correction, a difference in performance measures between models in bootstrap samples and models applied on original data was calculated.

All analyses were conducted using R version 3.6.1 [46] under application of the psfmi-package for model estimation and validation [47]. In the stepwise procedure, a liberal alpha-value of α = 157 was applied. For all other analyses, the significance was set at 5% (α = 0.05).

## 3. Results

### 3.1. Participants

The data set consisted of N = 1015 individuals (25% female), with a mean age of 44.20 years (±13.70), who participated in the assessments one year after TBI and who were admitted to the study hospital either to a ward or an ICU. Table 1 shows sample characteristics for dependent and independent variables (including factor levels) for original and imputed data (mean of five imputed data sets). The numbers in parentheses are used for further references in the text.

The majority (60.2%) experienced a mild TBI (GCS ≥ 13), followed by severe (24.7%; GCS ≤ 8), and moderate (11.3%; GCS 9–12) TBI. The mean GCS was 11.63 (SD = 4.33, IQR = 7). The average injury severity score was ISS = 24.56 (SD = 15.79, IQR = 21). In total, 68% had an ISS > 15, indicating a major trauma. Patients were admitted for on average two and a half weeks at the study hospitals (M = 16.94 days, SD = 24.97, IQR =18.28).

### 3.2. Employment Status

**Figure 2:**
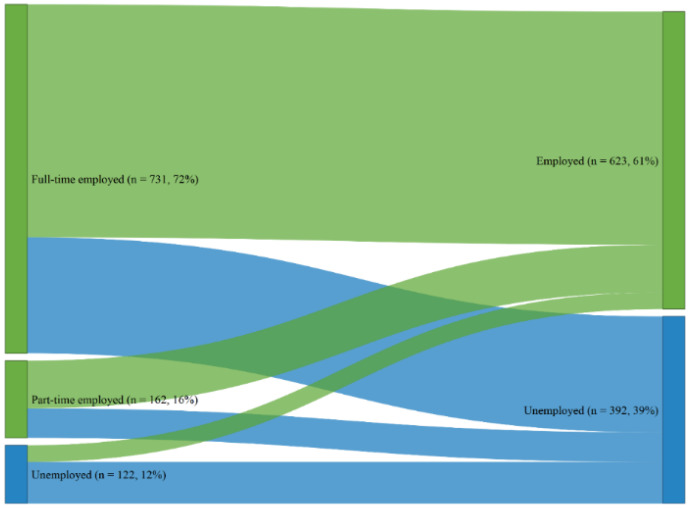
Employment status changes from baseline (left part) to one-year post TBI (right part). Note that the categorization of employment status at the two timepoints are different. At baseline employed: individuals were in labor market or currently at sick leave split in full-time and part-time groups; and unemployed: individuals were out of labor market. At one-year post TBI, employed individuals returned to work (either with the same amount of hours or with reduced hours, or individuals who changed the work); and unemployed individuals, who are unable to work and individuals looking for work.

Figure 2 shows change in employment status between baseline and one-year follow-up. Over the first year after TBI, the number of employed individuals decreased from 88 to 61%.

**Figure 3:**
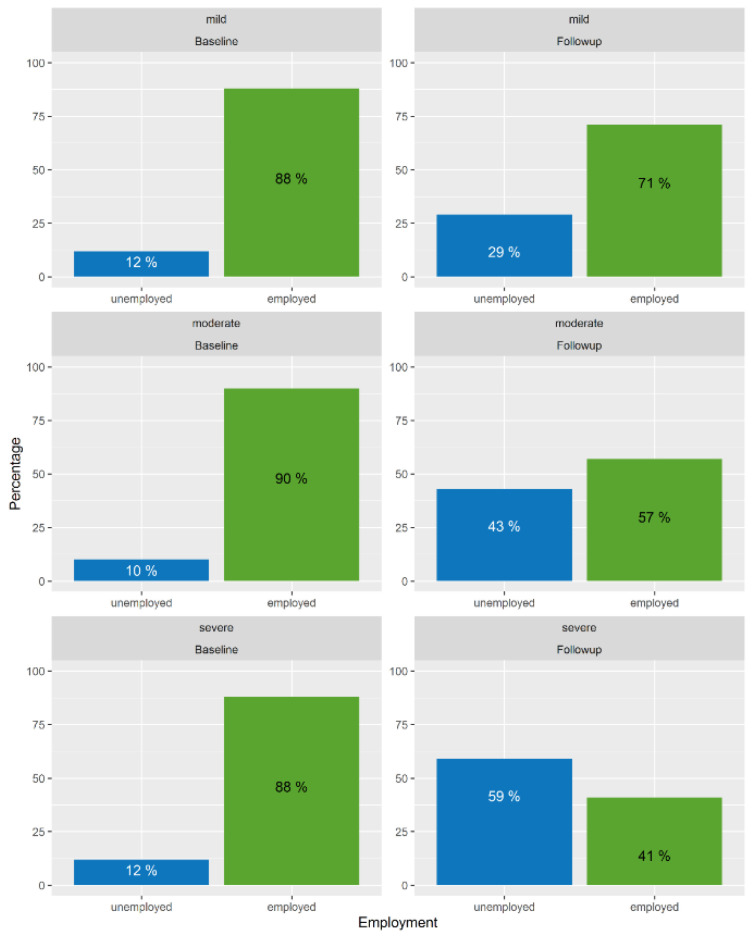
Employment rates at the baseline and one year after TBI by TBI severity groups.

Figure 3 visualizes the relative frequencies of employed individuals at the baseline and one year after TBI by TBI severity groups. At the baseline, 10–12 percent of individuals were unemployed. While the majority of individuals after a mild or moderate TBI (71% and 57%, respectively) returned to work one year after TBI, 59% of those suffering from severe TBI were unemployed.

### 3.3. Model Estimation and Stepwise AIC Procedure

The initial model estimation was based on the employment status at follow up as a dependent variable and factors derived from previous research (No. (1–12) in Table 1). The backward predictor selection revealed a simplified model which included the following sociodemographic, premorbid, and injury-related factors significantly influencing the probability of employment one year after TBI: age (2), employment status at baseline (5)*,* premorbid psychological/psychiatric problems (6), ISS (9), GCS score (10), and LOS at the hospital (12).

### 3.4. Final Model

**Figure 4:**
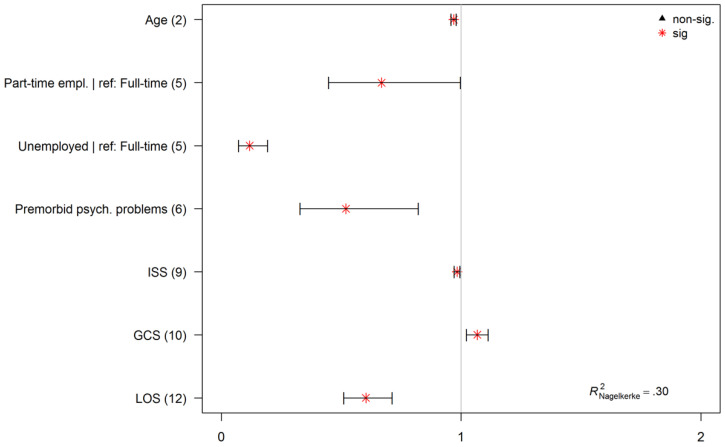
Odds ratios for the final model predicting probability of being employed one-year after TBI. Asterisks (*) indicate significant factors or factor levels. Values < 1 represent factors and factor levels with decreased probability of being employed one-year after TBI; values > 1 indicate for a higher probability of employment. Numbers in parentheses are referred to Table 1. The optimism-corrected Nagelkerke’s R² demonstrates the relative information gain of the final model compared to the null model (model without predictors, and intercept only).

According to the final model, five (2,5,6,9, and 12) out of six factors (higher age, less than full-time employment (part-time employment or unemployment at time of injury), higher ISS and longer LOS in the hospital) were significantly associated with decreased probability of employment one year after TBI and one factor (10) (higher GCS score) significantly increased the chance of being employed (see Figure 4 for visualization). For detailed results of the model estimation, see Table 2.

Sociodemographic factors. When keeping other factors constant, the probability of employment decreased by 3% per each one-year increase in age. Part-time employees and unemployed individuals (5) were also characterized by reduced likelihood of being employed one year after TBI compared to those working full-time at the time of injury.

Premorbid psychiatric history. Individuals suffering any psychiatric problems (6) prior to TBI were less likely to be employed one year after TBI.

Injury related factors. A higher total ISS (9) was associated with decreased probability of employment at the follow up. With one-point increase in ISS, the probability of employment decreased by 2%. With one-point increase in GCS score (10)*,* the employment probability 12 months after TBI increased by 7%. A longer LOS in the hospital (12) caused a significant decrease in employment probability 12 months after TBI.

Corrected Nagelkerke’s R² showed the value 0.30 (optimism correction of 0.02), indicating a prediction improvement from the null model to the final fitted model. The corrected AUC value was 0.79 (optimism correction of 0.01), which means that 79% of the employed individuals were classified as such in the model.

## 4. Discussion

The main purpose of this study was to determine early predictors associated with employment status one-year post-TBI in European countries. The results of this study emphasize the diminished employment rates among patients with TBI one-year post-injury and highlights some of the sociodemographic, premorbid, and injury-related factors associated with employment probability at this time point. Indeed, older age, status of part-time employment or unemployment at time of injury, higher injury severity (as measured with higher ISS, lower GCS, and longer LOS in the hospital), and premorbid psychiatric problems were all associated with increased unemployment probability at one year after injury.

In the present study, the rate of employment (61%) one-year post-injury was higher compared to the majority of studies that have been published previously. For instance, the most recent systematic review/meta-analysis on employment outcomes world-wide found an employment rate of 35% one year following moderate to severe TBIs [6]. In addition, the rate of employment was higher compared with those reported from other countries, such as the Netherlands (50% complete return to work [48]), Australia (56% considering competitively employed and paid work trial [13]), Norway (50% [15]; 55% [14]), and the United Kingdom (74% [49]; 60–75% [50]), and is higher compared to those reported from the USA based on the TBI model systems (range between 28% to 35% [19,34,51,52]), and lower compared to the reported rate from Taiwan (79% [27]).

The higher employment rates could be explained by methodological differences between the studies regarding injury severity, the definition of employment, and timing of assessment, among others. Regarding injury severity, in the current study, the majority of the sample had mild TBI (62.5%), followed by severe (25.7%), and moderate (11.8%) injury. Thus, it is not surprising that the rate of employment is higher than the majority of the studies mentioned above as they had higher percentages of individuals with moderate to severe TBI [19,34,51,52].

The definition and categorization of employment status vary across studies. For example, some researchers [21,34] have included students as “unemployed”, while others [14,15,53] have students included as “employed”. Additionally, some studies only include full-time competitive employment in the “employed” category [9], whereas others also include part-time employees [15]. Of course, as Sigurdardottir et al. [14] indicated, systemic factors are likely to be relevant, too, such as healthcare and insurance systems, cultural values, laws, and customs-related factors.

Studies may also vary according economic fluctuations related to the recruitment time-window (< 1990, 1990–2000, 2000–2010, and 2010–2020 decades). Across this period of time, different economic crises and recessions occurred (e.g., early 1990s recession, which affected Western countries; early 2000s recession, which impacted on the EU and the USA; 2007–2008 global financial crisis; 2009–2019 Eurozone crisis) that surely affected employment rates. It is well known that, during an economic recession, individuals with disabilities are a vulnerable group that may be affected negatively by layoffs and loss of employment opportunities [54]. This is correspondingly a concern with regards to the current severe acute respiratory syndrome coronavirus 2 (SARS-CoV-2) pandemic with huge global economic impact. In addition, a government’s passive and active employment policies and strategies may impact on the general population’s employment rates, as well as on the rates of individuals with disabilities. For example, the European Commission [55] developed the “European disability strategy 2010–2020” to empower individuals with disabilities to participate fully in the society and the economy. More recently, vocational rehabilitation programs are gradually implemented at rehabilitation centers, and this may improve employment rates in countries where these programs have been introduced. Lastly, advancements in technology available for most individuals (e.g., memory aids, as well as augmentative and alternative communication technologies) could also support individuals with TBI in fulfilling their duties at work.

Pre-injury employment status is one of the most consistent factors associated with employment one year after injury and later on, with a strong level of evidence reported in various systematic reviews [10,56]. As found within the current study, being unemployed at the time of injury has been linked to lower probability of employment one-year post-injury [13,21,24,57]. Furthermore, a recent study reported that the rate of job change after injury differs between individuals according to TBI severity from pre-injury to 12 months follow-up (19% of moderate/severe group; 12% of the mild group) [49]. It seems that not only the previous employment status is relevant but also prior type of occupation, with a consistent effect on employment rates regardless of educational level [26]. Those patients with previous professional and managerial positions (white collar) had the greatest probability of being employed one-year post-injury compared to manual labor occupations (blue collar) [15,18,26]. As the present study includes both employed and unemployed individuals, the usage of type of occupation as a predictor would lead to biased results as unemployed individuals could not be arranged to any type of occupation group.

The level of education has also been consistently associated with employment status in this population, with a strong level of evidence [10]. Several previous studies [21,26,34,51] have found that patients with less than high-school education showed a lower chance of employment post-injury. However, education was not found to be a significant predictor in the present study.

In accordance with the literature, sex was not associated with employment status, and marital status has been established as having a weak level of evidence [10]. Regarding age, the current study found a negative relationship with employment status, with older age increasing the probability of being unemployed. Although age has been reported with higher (younger age) or lower (older age) probability for employment after a TBI [11,13,14,27], the systematic review by Willemse-Van Son et al. [56] suggests an inconclusive level of evidence. Again, this pattern of relationship with employment status is not exclusive for individuals after TBI: in general, unemployed older workers have greater difficulties in finding a new job compared to unemployed younger workers [58]. Simultaneously, employment stability has been shown to be lower among younger individuals after TBI [18,59], perhaps due to less vocational skills and experience to aid in return to work (i.e., not yet achieved white collar level), lesser seniority if cut-backs in economic hardship, or merely due to a normal trend of increased job changes earlier in careers.

A history of premorbid psychiatric problems (i.e., anxiety, depression, sleep disorders, schizophrenia, drug abuse, or other psychiatric problems) significantly reduced employment probability one year after injury. Several conditions comprised in the variable in this study have been assessed as separate predictors in previous studies. For example, several studies demonstrated that pre-injury drug abuse is a significant predictor of unfavorable employment outcome [20,22,25,56,60]. Having a prior psychiatric diagnosis and receiving psychological treatment pre-injury have also been associated with reduced likelihood of employment post-TBI [22,61]. Moreover, a systematic review of acquired brain injury found evidence for increased risk of developing new psychiatric disorders in individuals with a history of psychiatric problems [62]. Thus, individuals who have experienced premorbid psychiatric problems may also have increased risk of psychiatric problems affecting work participation post-injury.

The results of the logistic regression analyses indicate that, among injury-related factors, total ISS, GCS, and LOS in the hospital are associated with employment status after a TBI one-year post-injury. LOS in the hospital has a strong level of evidence according to a systematic review [10], with patients who have shorter LOS in the hospital having greater chance of being employed. This is consistent with injury severity, measured in this study with ISS and GCS, given that individuals with less severe injuries usually have shorter hospital stays. Most studies have used GCS, presence of post-traumatic amnesia (PTA) or LOC, or the Disability Rating Scale (DRS) to measure injury severity, with results suggesting that patients with less severe injury (regardless of instrument used) have higher probability of being employed [13,14,15,18,21,27,34]. Interestingly, according to systematic reviews, while the level of evidence for GCS as a predictor of employment status in this population remains inconclusive [10,56], it seems that the level of evidence for DRS has changed from strong [56] to weak [10].

Chien et al. [27] found that ISS was more accurate at predicting employment status in their sample compared to GCS, Glasgow Outcome Scale (GOS) and Glasgow Outcome Coma Scale—Extended (GOSE) [63]. Perhaps ISS as an indicator of overall trauma severity is more sensitive to predict employment status at one-year post-injury, at least compared with GCS, as other injuries may affect return to work irrespective of the severity of the TBI. Functional outcomes, such as autonomy for activities of daily living or independence in transportation, may be better prognostic factors after TBI, as these variables (measured most of the time with the Functional Independence Measure) have been reported to be related with returned to work one-year post-injury [11,19,21,26,27,49].

Unlike previous TBI studies, injury cause was not significantly associated with employment status. Violent cause of injury has been related with higher odds of being unemployed one-year post-injury [21,34]. Interestingly, these studies have been conducted in the USA, where assaults are the fourth leading cause of TBI (10%), after falls (35%), motor vehicle crashes (17%), and blunt impact (17%) [64]. However, in Europe, the leading causes of TBI are falls and road traffic accidents [65]. In the present study, due to low prevalence, violence-related TBI was combined with other causes, which might explain the non-significant association with employment outcome.

### 4.1. Limitations and Strengths

Returning to employment after a TBI is a complex process that encompasses multiple individual, injury-related, and work-environment related factors. In this study, some of the factors, such as pre-injury or post-injury social support/employment support and others that have been reported previously as potential predictive factors, were not considered, such as cognitive functioning following injury [14,57], pre-injury health-related quality of life [49], self-awareness and self-monitoring [53], and occupational status [26], among others. The majority (60.2%) of patients in this study had a mild TBI, as assessed by the GCS (13–15). This is in line with other studies conducted across all TBI severity levels in patients admitted to trauma hospitals [65]. Thus, the rates of employment may be representative for individuals with both mild and moderate/severe TBI admitted to a hospital setting.

As a multi-country study across European countries, the present study is valuable as it confirms previous study results and strengthens the evidence base regarding which factors are important for the employment status at one-year post-injury. Single analyses for the respective European countries were beyond the scope of this study and are a topic for future ones.

It is common practice in research to exclude individuals with pre-existing psychiatric diagnoses from studies after TBI as the diagnoses may confound outcomes. However, this leads to a selection bias and a knowledge gap regarding outcome trajectories for individuals with TBI and associated premorbid psychiatric problems. Inclusion of these patients is thus a strength of the present study.

Finally, a promising relatively new aspect that may impact probability of employment at one-year post-injury is whether individuals are participating in a vocational rehabilitation program, as an increased support in the return to work process may impact employment rates. We did not analyze data on professional help and involvement in vocational rehabilitation among the present population, and this remains an area for future study. Interestingly, Radford et al. [50] found that more participants who underwent a vocational rehabilitation program returned to work, had more workplace adjustments, and felt greater employer support 12 months post-injury than those in usual care.

### 4.2. Implications

Taken together, unmodifiable factors, such as age, pre-injury employment, premorbid psychiatric problems, and injury severity-related characteristics, were predictive of employment status one-year post-TBI in this large, multi-country observational European cohort study. Vocational rehabilitation programs following TBI should take into account not only these factors but also possible modifiable work environment efforts, such as tailoring work tasks, supplying coaching in the workplace, ensuring workplace environmental support, flexible work hours, or graded work, etc. According to a recent systematic review on characteristics of work and workplaces that retain employees after acquired brain injury [66], this is an under-researched area with too few high-quality studies. To increase return to work among individuals after TBI, future methodologically strong studies should focus on work environment interventions that target modifiable work-related factors and better transition from long-term sick leave to work. Interestingly, the individual placement and support model has been shown effective in improving competitive employment rates among individuals with moderate-to-severe mental illness [67] and represents a relevant model for future investigation in the TBI population.

## 5. Conclusions

As part of the CENTER-TBI project, this study investigated some of the early predictors of employment status one year after mild-to-severe TBI among an adult population. The study found that older age, status of part-time employment or unemployment at time of injury, higher injury severity (as measured with higher ISS, lower GCS, and longer LOS in the hospital), and premorbid psychiatric problems were associated with higher unemployment probability at one year after injury. None of these factors are readily available for amelioration. Future studies need to focus on the development of interventions targeting modifiable work-related factors, tested in randomized controlled trials, to further increase employment rates after TBI.

## Figures and Tables

**Table 1:** Descriptive statistics for dependent and independent variables (factors and factor levels).

			Original Data			Imputed Data ^d^		
No	Variable	Groups (*Reference Group in Italic*) or No. of Cases	*N*	%	*M (SD)*	*N*	%	*M (SD)*
(1)	Sex	*female*	256	25.22%	-	-	-	-
		male	759	74.78%	-	-	-	-
		NA	0	0.00%	-	-	-	-
(2)	Age ^a^	valid cases	1015	100.00%	44.20 (13.70)	-	-	-
		NA	0	0.00%	-	-	-	-
(3)	Marital state	*partnered*	560	55.17%	-	-	-	-
		single	455	44.83%	-	-	-	-
		NA	0	0.00%	-	-	-	-
(4)	Education	*post-high school*	496	48.87%	-	535	52.71%	-
		secondary/high school	348	34.29%	-	382	37.64%	-
		none/primary school	90	8.87%	-	98	9.66%	-
		NA	81	7.98%	-	0	0.00%	-
(5)	Employment baseline	*full-time employed*	731	72.02%	-	-	-	-
		part-time employed	162	15.96%	-	-	-	-
		not working	122	12.02%	-	-	-	-
		NA	0	0.00%	-	-	-	-
(-)	Employment follow up	employed	623	61.38%	-	-	-	-
		*not working*	392	38.62%	-	-	-	-
		NA	0	0.00%	-	-	-	-
(6)	Premorbid psychological/psychiatric problems	*no*	881	86.80%	-	891	87.78%	-
		yes	122	12.02%	-	124	12.22%	-
		NA	12	1.18%	-	0	0.00%	-
(7)	Injury cause	*road traffic accident*	458	45.12%	-	459	45.22%	-
		fall	365	35.96%	-	366	36.06%	-
		violent/other ^c^	190	18.72%	-	190	18.72%	-
		NA	2	0.20%	-	0	0.00%	-
(8)	Stratum	*ward*	404	39.80%	-	-	-	-
		ICU	611	60.20%	-	-	-	-
		NA	0	0.00%	-	-	-	-
(9)	ISS score ^a^	valid cases	999	98.42%	24.56 (15.79)	1015	100.00%	24.63 (15.81)
		NA	16	1.58%	-	0	0.00%	-
(10)	GCS score ^a^	valid cases	977	96.26%	11.63 (4.33)	1015	100.00%	11.61 (4.34)
		NA	38	3.74%	-	0	0.00%	-
(-)	Injury severity ^b^	mild	611	60.20%	-	-	-	-
		moderate	115	11.33%	-	-	-	-
		severe	251	24.73%	-	-	-	-
		NA	38	3.74%	-	-	-	-
(11)	LOC	*no*	237	23.35%	-	272	26.80%	-
		yes	657	64.73%	-	743	73.20%	-
		NA	121	11.92%	-	0	0.00%	-
(12)	LOS ^a^	valid cases	994	97.93%	16.94 (24.97)	1015	100.00%	16.92 (24.88)
		NA	21	2.07%	-	0	0.00%	-
	Total		1015	100.00%	-	1015	100.00%	-

Note: Numbers in parentheses are used for indication in the text and further tables (except the dependent variable and the traumatic brain injury (TBI) severity groups); NA = not available, missing data; ^a^ for continuous variables and the total scores, mean (*M*) and standard deviation (*SD*) are reported. ISS = Injury severity score; GCS = Glasgow Coma Scale Score; LOC = loss of consciousness; LOS = length of stay in hospital; ^b^ TBI severity is computed as follows: mild TBI (GCS ≥ 13); moderate (9 ≤ GCS ≤ 12), and severe (GCS ≤ 8); ^c^ the group violent/other consisted of the following subgroups: other non-incidental injury (*n* = 57), violent/assault (*n* = 51), mass violence (*n* = 2), suicide attempt (*n* = 11), and other and unknown (*n* = 69); ^d^ data was imputed using the multiple imputation by a chained equations (MICE) procedure, mean values from five imputed data sets were calculated to provide descriptive statistics (reported only for variables used in the model with missing values).

**Table 2:** Results of logistic regression analyses.

No.	Variable/Category	Reference Group	Estimate	*S.E.*	*p*	Odds Ratios	CI_2.5%_	CI_97.5%_
(–)	Intercept	-	3.09	0.46	<0.001	22.004	8.9239	54.256
(2)	Age	-	−0.03	0.01	<0.001	0.97	0.96	0.98
(5)	Part-time employed	Full-time employed	−0.40	0.20	0.048	0.67	0.45	1.00
	Not working	Full-time employed	−2.14	0.25	<0.001	0.12	0.07	0.19
(6)	Premorbid psychiatric problems	No	−0.65	0.23	0.005	0.52	0.33	0.82
(9)	ISS	-	−0.02	0.01	0.007	0.98	0.97	1.00
(10)	GCS score	-	0.06	0.02	0.003	1.07	1.02	1.11
(12)	Length of stay (*ln*)	-	−0.51	0.09	<.001	0.60	0.51	0.71

Note: Numbers in parentheses are used for indication in the text and are referred to the Table 1; Estimate = logistic regression coefficients, *S.E*. = standard error, *p* = *p*-value, Odds ratios = odds of being unemployed in relation to the odds of being employed, CI_2.5%_= lower bound, CI_97.5%_= upper bound, and *ln* = the variable length of stay was transformed because of the skewed distribution.

## Supplementary Materials

The following are available online at https://www.mdpi.com/2077-0383/9/6/2007/s1, The list of CENTER-TBI participants and investigators.
